# A New Graph Drawing Scheme for Social Network

**DOI:** 10.1155/2014/930314

**Published:** 2014-07-16

**Authors:** Eric Ke Wang, Futai Zou

**Affiliations:** ^1^Shenzhen Key Laboratory of Internet Information Collaboration, Shenzhen Graduate School, Harbin Institute of Technology, Shenzhen 518055, China; ^2^School of Information Security Engineering, Shanghai Jiaotong University, Shanghai 518055, China

## Abstract

With the development of social networks, people have started to use social network tools to record their life and work more and more frequently. How to analyze social networks to explore potential characteristics and trend of social events has been a hot research topic. In order to analyze it effectively, a kind of techniques called information visualization is employed to extract the potential information from the large scale of social network data and present the information briefly as visualized graphs. In the process of information visualization, graph drawing is a crucial part. In this paper, we study the graph layout algorithms and propose a new graph drawing scheme combining multilevel and single-level drawing approaches, including the graph division method based on communities and refining approach based on partitioning strategy. Besides, we compare the effectiveness of our scheme and FM^3^ in experiments. The experiment results show that our scheme can achieve a clearer diagram and effectively extract the community structure of the social network to be applied to drawing schemes.

## 1. Introduction

Graph drawing is a combination technique of information science and mathematics, which is employed in multiple research areas such as social network analysis. Since social networks are commonly very complex in large amount of data about features and relationships, it is difficult for people to understand the huge data. Fortunately, graphs help analytics in visualization and rationalization. Graph drawing is, given a set of nodes and sets (edge sets) of their relationships, to calculate the position of each node and plot the edges as curves. In other words, it is a transforming way from abstract data such as text and digits to static or dynamic visualized results in order to let people easily understand the principle and inner meaning of huge amount of complex data. It helps people make judgmental and analytic decision from the macro view. But, although graph drawing for social networks has been studied for several years, there are still many problems to be solved.

Currently, in most schemes of graph drawing, one social network is regarded as one kind of community structure to draw the graph instead of multiple communities in one social network. However, a social network commonly has possible features of various communities. Thus, it leads to an appearance that many graph drawing algorithms can perform well in some data sets with certain features but perform badly in more complex data. Therefore, how to detect various community structures in social networks and adapt drawing to current structure are important research problems to be solved.

Besides, in many data sets of social networks, there are various semantic information fusions and exchanges among members; however, in current drawing approaches, the impact of visualization for the semantic information is not considered; only topology model or structure features are employed. Thus, it may result in many graphs being unreadable and readers hardly fully extract the information of members or communities they care about from the drawn graphs. Therefore, we need a new drawing approach which combines topology and semantic information to make the drawn graphs readable and reasonable.

## 2. Related Works 

Currently, there are mainly several categories of graph drawing approaches such as node-link [1–4], space filling [5, 6], matrix [7–9], and mix [10, 11]. Node-link is relatively simple, considering nodes as vertices, only calculating their positions and representing edge as curve or fold line; space filling is a reduction of multidimensional problems, for example, reducing 3-dimensional problems into 2-dimensional problems. A nested curve such as Hilbert m-Peano curve is recursively refined to represent the data. Matrix approach represents a diagram as a connected matrix, (*i*,  *j*) is represented as the edge from node *i* to node *j*, and the attributes of the edge are encoded in visual features such as color, form, or size.

For node-link approach, there are two main drawing algorithms (single-level drawing algorithm and multilevel drawing algorithm).

In single-level drawing approaches, there are several typical types such as tree based [12], radical [13], and force directed [6]. Among them, force directed is widely used for drawing. The idea of force-directed way is proposed by Eadges [14]. It is that, mapping the relationships into physics mechanics models, nodes are replaced by small solid balls with some certain radius; the edges are replaced by springs. In initialization, the coordinates of each small ball are generated randomly. And then, to use the elastic force of springs on the balls to move the positions of the balls until the energy of whole system is minimal at last is what we call optimal state. Lately, the spring algorithm is updated in several schemes [2] and [15–17]; the major difference among them is the way to compute elastic force.

Multilevel scheme is mainly used to improve the effectiveness of layout and shorten drawing time. The main idea of multilevel scheme is to recursively apply the coarsening of diagrams. The coarsening of diagrams is the abstraction representation of fine-grained diagrams in multilevel and it can be drawn much faster. In other words, it can be applied for larger data sets to enhance the visualization effectiveness and reduce the running cost at the same time. FM^3^ (fast multipole multilevel method) [15] is a classical multilevel algorithm applying for most of the graphs. In FM^3^, the diagram is segmented to several child diagrams called “solar systems”; each “solar system” is compressed to be a node and repeats the process until forming a hierarchical diagram. The method had a better effectiveness than former approaches [18]. Walshaw [19] proposed a kind of evaluation method to coarsen by maximum matching. Maximum matching is a greedy algorithm to contain the largest possible number of edges. ACE algorithm [20] decides the number of partitions by solving Laplacian matrix. The feature vectors are computed by constructing a hierarchical coarsening matrix and recursively evaluating the feature vector of each level to achieve the vector of the original diagram. Archambault et al. [21] proposed a multilevel approach based on topological feature. In the approach, the interested topological feature is firstly detected and the child diagram with the topological feature is replaced with a node in the coarsening level. And then recursively execute detection for the features and compression process. In the process, for the topological feature of each child diagram, a proper drawing approach is selected.

In this paper, we propose a new drawing scheme combining multilevel drawing and single-level drawing approaches, including the graph partition method based on communities and layout refining approach based on partitioning strategy. Graph partition based on communities is employed in the stage of graph division of multilevel drawing. Single force-directed algorithm is used for the setting of initial coordinates of layout refining process; the layout refining process based on partitions is used for the iteration of initial coordinates and optimizing process to achieve the best layout effectiveness. Besides, we compare the effectiveness of our scheme with FM^3^ in experiments. The experiment results show that our scheme can achieve a clearer diagram and effectively extract the community structure of the social network to be applied to drawing algorithm.

The objective of our graph drawing scheme is to quickly present readable graphs to the users which can also precisely reflect the data principle. It mainly includes three goals:

1. recognizing the communities accurately,
2. adaptive layout,
3. reasonable use the layout space to reflect the strong and weak relations among vertices.

We propose a new adaptive scheme to achieve the above goals.

## 3. Assumption and Notations

In this paper, we mainly research on undirected graphs. Undirected graph can be represented by *G* = {*V*, *E*}, where *V* represents the set of vertices and *E* represents the set of edges. In our work, we target on connected undirected graph. The definitions of notations are as follows:

 |*V*| is number of vertices;
 |*E*| is number of edges;
 
*r*(*v*) is neighbor set of node *v*;
 
*e*(*i*,  *j*) is the edge between nodes *i* and *j*;
 Pos(*x*_*i*_) are the coordinators for distributing nodes;
 ||*x*_*i*_ − *x*_*j*_|| is the distance between two nodes in the graph;
 
*d*(*i*,  *j*) is the distance between any two nodes.

In our scheme, we adopt small-world network theory. As we all know, researchers have studied small-world network theory for a long time, but most of the researches focus on exploring the principle and topology of small-world networks. For example, a common job of social networks analysis is recognition of the modes and relations among the connected nodes which represent some social implications such as social status. Actually, many networks to be visualized have some features such as community characteristics. Those features can be easily recognized by people straightforwardly if they are shown in a graph. However, most of researchers focus on data and topological analysis of social networks, while, in the area of information visualization, small-world network theory is not fully employed. Therefore, we propose a graph drawing scheme based on small-world network theory. We separate a network to some small hierarchical communities which are highly connected with each other inside each community. And it is much more convenient for users to observe the relations and groups among members and understand the relations structures in the graph.

## 4. The Graph Drawing Scheme

It mainly involves two steps: (1) communities partition and (2) adaptive refinement.

### 4.1. Communities Partition

**Figure 1:**
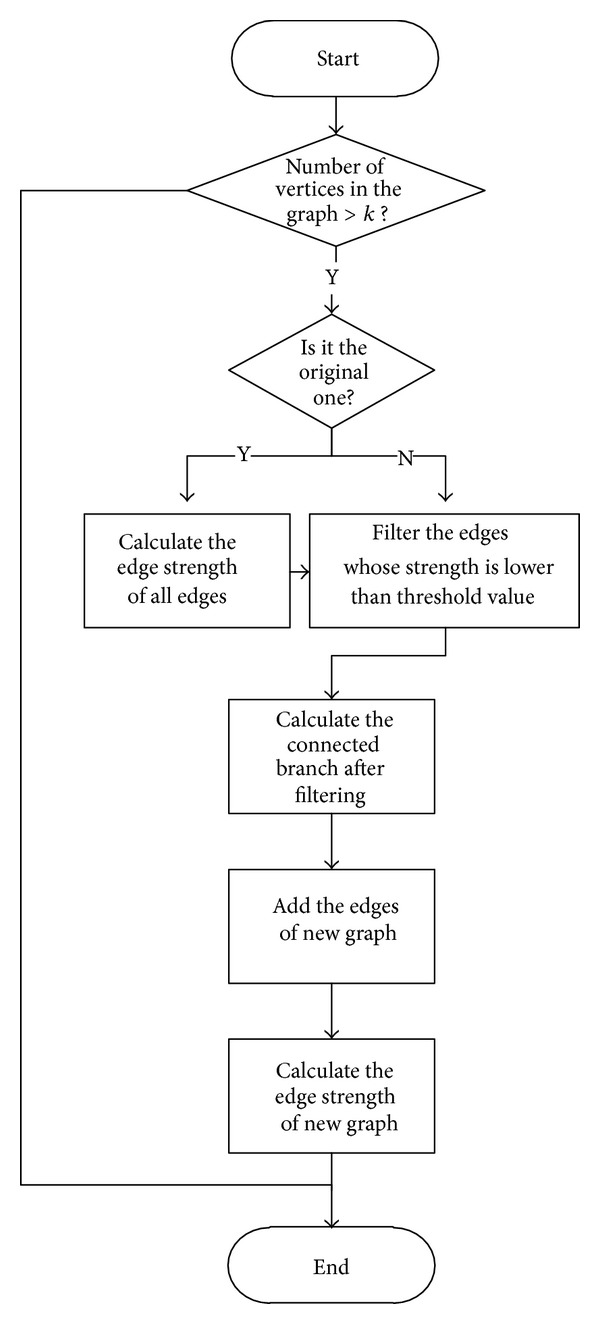
Flow of layout compression.

In community partition, we adopt a filtering approach to separate a graph into a hierarchy of subnetworks by finding out the weakest edges as the separation starting edges. The procedure is as shown in Figure 1.

The process is to calculate the edge strength to find out the weakest edges in the network and then delete the weakest edges so that it can be separated into subnetworks with stronger connections inside each subnetwork.

The procedure of community partition can be divided into 3 steps.

(*1) Filter Out Weak Edges.* The edge strength represents its contribution to the clustering coefficient. If an edge connects two uninteracted groups of neighbors, then strength of the edge is considered* zero*. Thus, the edge is weak and filtered out.

We can set up a threshold value *τ*, and once strengths of the edges are lower than *τ*, they would be filtered out. Thus, the original graph can be divided into some subnetworks. Based on our observation, we found that the threshold value *τ* is related to the maximum of edges strengths instead of empirical value. Then we propose an approach to identify the threshold value. Find out the maximum and minimum of the strengths of all edges, and then *τ* = min_ES_ + (max_ES_ − min_ES_) × ratio). When ratio is close to the biggest strength such as 0.95, it can guarantee the accuracy.

**Figure 2:**
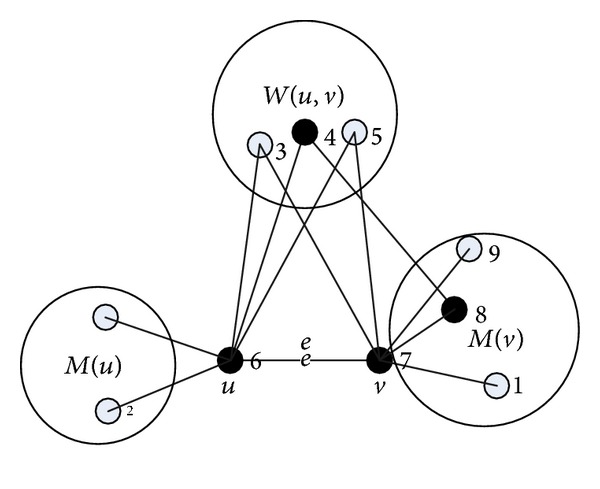
*e*(*u*, *v*) and neighbors.

Given an edge *e*(*u*, *v*), the edge strength can be calculated as follows (as shown in Figure 2).

(1) Separate the neighbors of *u* and *v* into three subsets which have no interaction with each other.
(2) *M*(*u*) represents the set of all *u*'s neighbors which are not adjacent to *v*.
(3) Similarly, *M*(*v*) represents the set of all *v*'s neighbors which are not adjacent to *u*.
(4) *W*(*u*,  *v*) represents the set of common neighbors of *u* and *v*. *r*(*A*, *B*) represent the number of edges between set *A* and set *B*.
(5) *S*(*A*, *B*) = *r*(*A*, *B*)/(|*A* | ∗ | *B*|) represents the ratio of the real exist edges and all possible edges between *A* and *B*.
(6) Any edge between *M*(*u*) and *M*(*v*) or *W*(*u*, *v*) is a part of a 4-edge closed loop which must have an edge of *e*(*u*, *v*).
(7) We define that |*W*(*u*, *v*)|/(|*M*(*u*)|+|*W*(*u*, *v*)|+|*M*(*v*)|) is the ratios of 3-edge closed loop including *e*(*u*, *v*). Then the strength of *e*(*u*, *v*) can be calculated by the following equations:
  (1) ES(u,v) =s(M(u),W(u,v))+s(W(u,v),M(v))+s(W(u,v))  +s(M(u),M(v))+|W(u,v)||M(u)|+|W(u,v)|+|M(v)|.

*(*2)* Hierarchical Decomposition*. Recursively apply filtering out weak edges until the final graph has enough small size to maintain the communities characteristics.

*(*3)* Selection of Threshold Value* *τ Decides the Form Way of Clusters.* The technique of graph drawing provides a good view way for the data sets. When graph becomes very big, subgraphs would be presented by dense areas.

However, this kind of hierarchical clustering way has a disadvantage which is that deciding the edges strength of higher level graph is difficult after each time of hierarchical decomposition. For example, there are three subgraphs *H*_*i*_, *H*_*j*_, and *H*_*k*_ which are the same size. In the original graph, there were 10 edges between *H*_*i*_ and *H*_*j*_, while there are 2 edges between *H*_*j*_  and *H*_*k*_. Apparently, the relation between *H*_*i*_ and *H*_*j*_ is much closer than that between *H*_*j*_ and *H*_*k*_. But, after the hierarchical decomposition, in the high level graph, the number of edges between *H*_*i*_ and *H*_*j*_ becomes 1, so does the number of edges between *H*_*j*_ and *H*_*k*_. It completely loses the weight of relations. It is not reasonable. Therefore, we propose an approach to solve the problem.

Suppose the original graph is *G*_0_, after a time of hierarchical clustering, a higher graph *G*_1_ is generated, and then *G*_2_ and *G*_3_ are generated sequentially. Suppose *e*(*u*, *v*) is one edge of *G*_0_ and its strength is smaller than *τ*; that means that in the clustering process it would be deleted. After it is deleted, *u* and *v* are located in two different clusters *C*_*u*_ and *C*_*v*_; we use *H*_*u*_ and *H*_*v*_ to represent two clusters. Then, in the higher level graph, there would be an edge *e*(*H*_*u*_, *H*_*v*_) which connects *H*_*u*_ with *H*_*v*_. Then, in *G*_1_, the new edge strength is calculated as the following formula:

(2) ES(Hu,Hv) =1nCu,Cv∑e(u′,v′)ES(u′,v′) u′∈Cu,  v′∈Cv.

At the same time, *n*_*C*_*u*_,*C*_*v*__ represents the number of edges between *C*_*u*_ and *C*_*v*_ in *G*_0_. That means the edges strength in *G*_1_ is decided by *G*_0_.

### 4.2. Adaptive Refinement

In graph drawing of social networks, analyzers are often interested in the topology of relationships and consider drawing strategy according to physical model, instead of the semantic information. Thus, it leads to much important relationship information which would be covered or lost. Therefore, we propose a new refinement approach to fully use layout space to make the visualization result intuitionistic based on topology and semantic characteristics. It mainly achieves three objectives: (1) make the communities partition as clear as possible by regionalization that is making drawing of the different communities in different regions, and the size of regions is expected to reflect the size of communities; (2) make full use of layout region which can minimize the intersection part of each of subregion; (3) decide the distance between two vertices which is expected to reflect their relation strength, and the distance between two communities is also expected to reflect two communities relation strength. To achieve the three goals, the approaches are as follows.

Suppose there are constructed hierarchical graphs list List *G* = {*G*_0_, *G*_1_,…, *G*_*i*_}, *i* = 0,1, 2,…, *k*, for graphs *G*_*i*_ and *G*_*i*+1_, where *G*_*i*+1_ is compressed result from *G*_*i*_. Suppose *G*_*i*_ has *N*_*G*_*i*__ nodes, *N*_*C*_*u*__ is the size of the community *C*_*u*_ nodes set, *u* is one vertex of *G*_*i*+1_ which is compressed result from *C*_*u*_ of *G*_*i*_, Area_*u*_ is the distributed area of *u* in the process of layout for *G*_*i*+1_, and Rect(*u*) is the rectangle area of *u* in the limited area of *G*_*i*+1_. The area of each vertex of the layout for *G*_*i*+1_ is calculated as follows.

**Figure 3:**
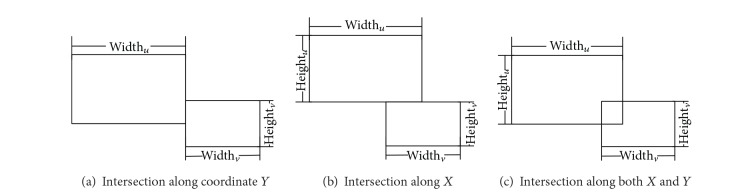
Status of intersection of rectangles.

(1) Calculate the area of *u*:
  (3) $$\begin{array}{c} \text{Area}\left(u\right)=\frac{N_{C_{u}}}{N_{G_{i}}}\times \text{Are}{\text{a}}_{G_{i+1}}, \end{array}$$
(2) Calculate the rectangle area of *u* partitioned in the layout of *G*_*i*_, and the width and height of Rect(*u*) should satisfy the equations:
  (4) $$\begin{array}{c} \text{Rect}\left(u\right)=\text{Width}\left(u\right)\times \text{Height}\left(u\right)=\text{Area}\left(u\right),\\ \frac{\text{Width}\left(u\right)}{\text{Height}\left(u\right)}=\frac{\text{Width}\left(G_{0}\right)}{\text{Height}\left(G_{0}\right)}. \end{array}$$
  Since there maybe overlaps between rectangles, we need to reduce the overlaps.
(3) Locate the subrectangle and minimize the overlap of rectangles. In other words, reducing the overlap area represents more usage of space. After the step (2), get the rectangle areas of all the vertices of *G*_*i*+1_; we need to distribute them to appropriate positions to make the overlap minimum. For human view, each subrectangle can be laid flat on the original rectangle. The overlap areas of subrectangles are calculated as follows (shown in Figure 3).

Suppose *u* and *v* of *G*_*i*+1_ represent two subrectangles, their areas are marked as Rect(*u*) and Rect(*v*), their width and height are width_*u*_ and width_*u*_ and height_*u*_ and height_*v*_, and the coordinates of their center points are *P*(*u*) = (*x*_*u*_, *y*_*u*_) and *P*(*v*) = (*x*_*v*_, *y*_*v*_). Consider *P*(*u*)_*x*_ = *x*_*u*_ and *P*(*v*)_*x*_ = *x*_*v*_. To identify whether two rectangles have intersection area, we need to check whether the distance between two center points is bigger than half of the sum of their width or height along the coordinates *X* or *Y*. The following equations should be satisfied:

(5) $$\begin{array}{c} \left|P{\left(u\right)}_{x}-P{\left(v\right)}_{x}\right|\leq \frac{1}{2}\left(\text{widt}{\text{h}}_{u}+\text{widt}{\text{h}}_{v}\right),\\ \left|P{\left(u\right)}_{y}-P{\left(v\right)}_{y}\right|\leq \frac{1}{2}\left(\text{heigh}{\text{t}}_{u}+\text{heigh}{\text{t}}_{v}\right). \end{array}$$

If the above two equations are satisfied, two rectangles would have intersection part.

After identifying that they have intersection part, we can calculate the overlap area as the following formula:

(6) Area(Rect(u),Rect(v)) =|P(u)x−P(v)x|×|P(u)y−P(v)y|.

After computing the overlap areas of all subrectangles, we need to maintain a matrix 0-1 to record whether they are intersected with each other. Then we need to check them one by one; if there are *N*_*G*_*i*+1__ vertices in the graph *G*_*i*+1_, we need to maintain a 0-1 matrix whose size is *N*_*G*_*i*+1__ × *N*_*G*_*i*+1__, marked as Shadow*M*. Then all overlap areas can be calculated as the following formula:

(7) $$\begin{array}{c} \overset{N_{G_{i+1}}}{\underset{i=1}{\sum }}\;\overset{N_{G_{i+1}}}{\underset{j=1}{\sum }}\text{Shadow}M\left(i,j\right)\times \text{Area}\left(\text{Rect}\left(u\right),\text{Rect}\left(v\right)\right). \end{array}$$

Then, according to the objectives, we need to make the overlap areas as small as possible.

In order to achieve the third goal, we define the standard distance between two entities: suppose the edge strength of two entities is ES(*e*), and then their standard distance is calculated by the following formula:

(8) $$\begin{array}{c} d_{u,v}=\frac{1}{\text{ES}\left(e_{uv}\right)}. \end{array}$$

Suppose *P*(*u*) = (*x*_*u*_, *y*_*u*_) and *P*(*v*) = (*x*_*v*_, *y*_*v*_) are the actual coordinates of vertices *u* and *v*, and then the real distance ${d_{}^{\prime }}_{u,v}=1/\sqrt{{\left|x_{u}-x_{v}\right|}^{2}+{\left|y_{u}-y_{v}\right|}^{2}}$; we use Δ*d*_*u*,*v*_ = *d*_*u*,*v*_ − *d*′_*u*,*v*_ to represent the deviation of real distance and the edge strength. So Δ*d*_*u*,*v*_  is expected to be as small as possible to make sure the real distance can reflect the edge strength.

Considering the three above goals, the objective function is as follows:

(9) F=∑i=1NGi+1 ∑j=1NGi+1(ShadowM(i,j)×Area(Rect(u),Rect(v))+Δdu,v).

## 5. Main Algorithms

In our scheme, we propose three main algorithms:

1. community partition algorithm,
2. hierarchical compression algorithm,
3. optimization algorithm based on blocks.

The first one is used to compress the first layer; the second algorithm is for generating hierarchical compression map which is refined in the third algorithm.

### 5.1. Community Partition Algorithm

Community partition algorithm is the first step of our scheme. The procedure is as follows.

1. Calculate the set of neighbors of all edges, including the single neighbor-sets of the two nodes of one edge which have no intersection part, 3-edge circle neighbor-sets which can form a circle including 3 edges between the neighbor and the two nodes, and 4-edge circle neighbor-sets which can form a circle including 4 edges between the neighbor and the two nodes. These neighbor-sets are the base of calculating the strength of edges in the next step.
2. Calculate the strengths of the above edges.
3. Compare and find out the maximum and minimum of edge and calculate the filter threshold value.
4. Delete all the edges whose strengths are lower than threshold value and update the diagram.
5. Recalculate the connected components of updated diagram and return the value.
6. Compress each connected component into a packed node as a new vertex of the updated graph.

And we add edges for new vertices if they have relationship in their original diagrams.

The pseudocode is shown in Algorithm 1.

### 5.2. Hierarchical Compressions

Hierarchical compression is the second step; its pseudocode is shown in Algorithm 2.

The algorithm is built on algorithm 1. It firstly compresses the original diagrams and puts them into a stack, and then judge whether the top element of queue satisfies the compression condition; if yes, take it out, compress the front element, and push it into the queue again.

### 5.3. Optimization Algorithm Based on Blocks

The main procedure of single-level partition algorithm includes three steps:

1. adopt spring algorithm to locate the coordinates of single-level diagram,
2. set partitions and put each vertex into the partitions,
3. execute iteratively gradient descent to achieve the minimum of intersection of the partitions.

The algorithm is a loop procedure which processes the diagram of each layer. In the first step, it adopts spring to locate the initial positions of single-level diagram; secondly, it traverses all the vertices; because each vertex corresponds to the new child diagram connected component from the top of the stack, the size of each component is different from the others. Then, according to the size of component, it allocates subareas which are in proportion to the width and height of original area, and set the center coordinate as the initial coordinate of the vertex from the first algorithm. In the third step, based on iteratively gradient descent approach for the intersected parts of subareas, the minimum value can be achieved and the final coordinates of all areas are created. Its pseudocode is shown in Algorithm 3.

## 6. Evaluation

In community partition, we compare our scheme with a popular partition approach [3] which is based on empirical value. We adopt 10 data sets of social networks with communities' structure. The configuration of experiment is operating system, WIN7, CPU, Intel(R) Core(TM)4 Quad CPU2.33 GHZ; Memory, 4 G, and area of layout is 1500∗850.

In multilevel drawing stage, we compare our scheme with fast multilevel algorithm FM^3^. The data sets are from Newman classical data sets which include two groups of artificial social networks graphs with communities, three real data sets “subScience,” “football,” and “polbooks.” The artificial graphs include a social network with 128 nodes and 1009 edges and a scientists working network with 379 nodes and 914 edges. Football graph represents the networks of competitions which involve the football teams in a competition season, which include 114 nodes (teams) and 615 edges (competitions); polbooks is the data set of the sales of American political books in http://www.amazon.com/ where edge represents that two books are bought together in one order.

### 6.1. Evaluation Metrics

We adopt cluster quality value MQ to evaluate the accuracy of community partition. MQ is the average value of the density of edges inside a community. After partition for a graph, if MQ is bigger, it represents that the partition result is closer to real community result. MQ is calculated as follows:

(10) $$\begin{array}{c} \text{MQ}\left(C,G\right)=\frac{1}{p}\overset{n}{\underset{i=1}{\sum }}S\left(C_{i},C_{i}\right)-\frac{1}{p\left(p-1\right)/2}\overset{}{\underset{i<j}{\sum }}S\left(C_{i},C_{j}\right). \end{array}$$

In several common algorithms, the selection of MQ threshold value is decided by empirical value based on statistics approach. Given a value of MQ as “*c*,” we can identify the probability of partition effect higher than *c*.

In our scheme, the threshold value is computed by *τ* = min_ES_ + (max_ES_ − min_ES_) × ratio), which is described in Section 4.1.

### 6.2. Community Partition Comparison

According to 10 social networks data sets, we compare our scheme with the threshold approach in cluster quality value; the result is as shown in Table 1.

From Table 1, the MQ value based on empirical value is less than MQ of our scheme. We can find that the average value of MQ based on empirical value is smaller than that of MQ based on threshold value selection approach of our scheme.

### 6.3. Comparison of Layout Effects

**Figure 4:**
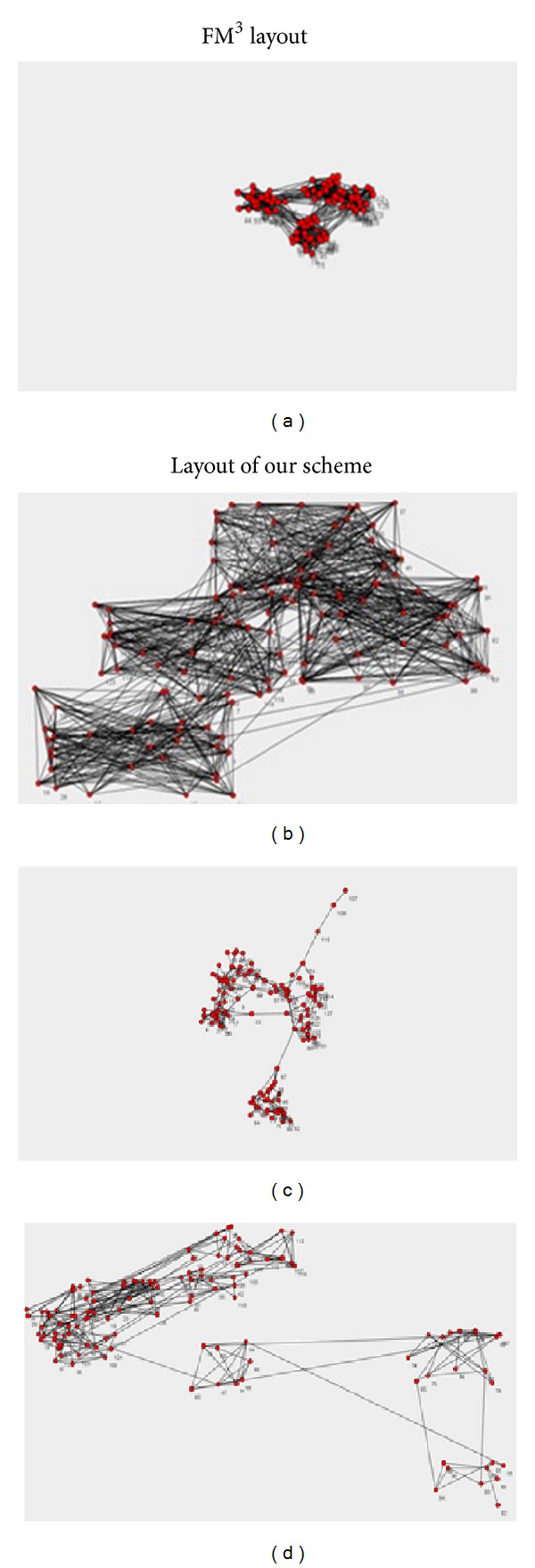
Comparison of community discovery for our scheme and FM^3^.

Figure 4 shows the drawing effectiveness comparison of FM^3^ algorithm and our algorithm on the artificial social networks data sets. The first data set has 128 nodes and 1009 edges and four obvious communities; the second data set has 5 obvious communities.

From Figure 4, our scheme and FM^3^ can both recognize four communities in the graph; the difference is that FM^3^ often presents the whole layout in a very small region; the communities partition is clear but the degree of overlapping inside the communities is high; the main reason is that the configuration of parameters needs a lot of experiments and validations to adjust the empirical values for various kinds of layout regions. Therefore, if operators are not familiar with the algorithms or do not have enough experience, they need much longer time to adjust experiments to achieve an ideal drawing result. However, in our scheme, not only four communities are recognized correctly, but also the layout space is better for visualization. Besides, it only needs one parameter to be configured (the percentage of users' expectation on final layout space). Thus, it is easy for users to understand and operate.

**Figure 5:**
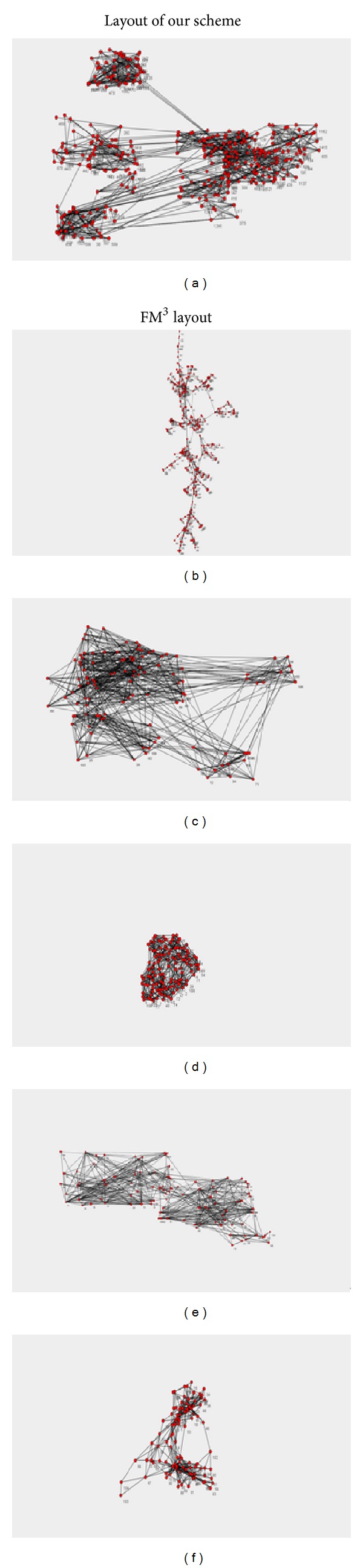
Comparison of layout effectiveness between our scheme and FM^3^.

Figure 5 shows the comparison of the drawing results between our scheme and FM^3^ on three groups of real data sets with some certain community structures. The first data set is a researchers collaboration relationship graph, “subScience,” with 379 vertices and 914 edges; the second data set is a competition schedule graph of a football club, “football,” with 114 vertices and 616 edges; the third is the sales situation of books about American Politics on http://www.amazon.com/, “polbooks,” with 105 vertices and 882 edges.

For the first data set, our scheme can recognize the main community's structures and make full use of layout space; for the second, our scheme can present the distribution of competitions of teams; for the 3rd data set, our scheme can partition 3 categories of buyers and make good use of layout space. In general, our scheme has better layout effects than FM^3^.

## 7. Conclusion

We studied graph drawing schemes and propose a new scheme for social networks which improves the graph drawing effectiveness. Besides, we compare the effectiveness of our scheme with FM^3^ in experiment. The experiment result shows that our scheme can effectively extract the community structure of the social network to apply into drawing algorithm and achieve a clearer diagram.

## Figures and Tables

**Algorithm 1:**
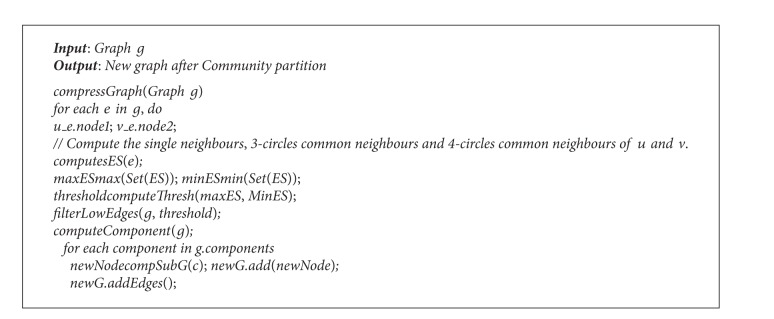
Community partition algorithm.

**Algorithm 2:**
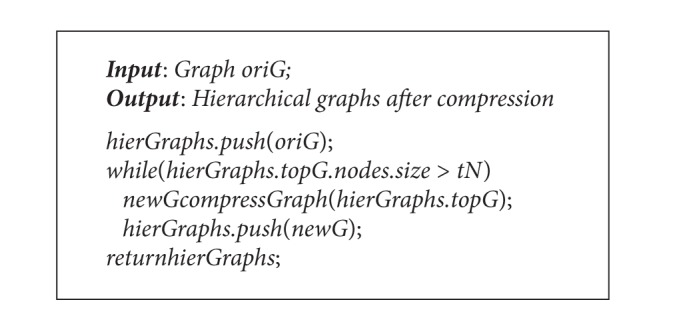
Hierarchical compression algorithm.

**Algorithm 3:**
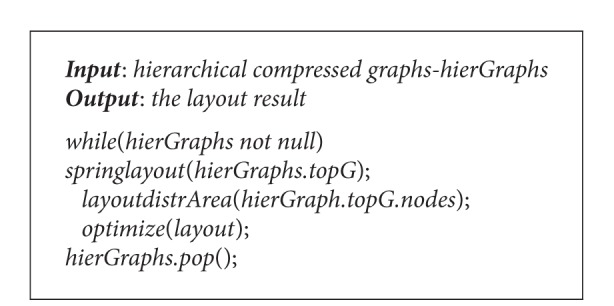
Optimization algorithm based on blocks.

**Table 1:** Threshold value and MQ on several data sets.

Graph	Threshold value of our scheme	MQ	Empirical value	MQ based on empirical value
128 my	2.34	−2042	1.75	−52
128	2.82	−536	1.95	−206
Football	2.5	−1261	1.84	−241
Polbooks	3.045	−894	2.27	−734
SubScience	3.03	−1882	2.22	−867
